# The Ipsilateral Interhemispheric Transprecuneal Approach to the Atrium: Technical Considerations and Clinical Outcome on a Series of 7 Patients

**DOI:** 10.3390/brainsci12111453

**Published:** 2022-10-27

**Authors:** Lorenzo Pescatori, Maria Pia Tropeano, Fabio Torregrossa, Giovanni Grasso, Pasqualino Ciappetta

**Affiliations:** 1 Department of Neurosurgery, Sant Eugenio Hospital, 00144 Rome, Italy; 2 Humanitas Clinical and Research Center-IRCCS, 20089 Milan, Italy; 3 Department of Biomedical Sciences, Humanitas University, 20090 Milan, Italy; 4 Neurosurgical Unit, Department of Biomedicine, Neurosciences and Advanced Diagnostics (BiND), University of Palermo, 90133 Palermo, Italy; 5 Department of Neurosurgery, University of Bari, 70121 Bari, Italy

**Keywords:** intraventricular tumor, interhemispheric approach, surgical management, atrium, superior longitudinal fasciculus

## Abstract

Surgical removal of tumors of the atrium is challenging due to their deep location, vascularization, and to their complex three-dimensional relationships with the highly functional white matter fibers of the region. To assess the feasibility and the effectiveness of the ipsilateral interhemispheric transprecuneus approach (IITA) for tumors involving the atrium and the posterior third of the temporal horn, a retrospective chart review of all patients who had undergone a surgical treatment for intraventricular tumors between January 2008 and January 2017 was performed, and the step-by-step approach is described. Ten patients affected by lesions of the atrium of the lateral ventricle underwent surgical treatment, seven of which were approached through the IITA. The mean age was 42.8 years (range 6–63 years). The symptoms presented included severe, drug-resistant headache (90%), lateral homonymous hemianopsia (50%), seizures (30%), and speech disturbances (30%). Histological examinations revealed seven patients with meningioma (70%), one with a metastasis (10%), one with a choroid plexus papilloma (10%) and one with a cavernoma (10%). In all cases, a gross total removal was obtained. All patients had a significant improvement in their headache. Two patients experienced a worsening of the pre-operative visual disturbances, while two patients had a significant improvement. No patients without pre-operative visual disturbances described a post-operative worsening of visual symptoms. The IITA represents a feasible approach for tumors of the atrium. The three-quarter prone position facilitates the enlargement of the interhemispheric fissure by increasing the working angle and facilitating the exposure of the lateral wall of the atrium.

## 1. Introduction

**Figure 1:**
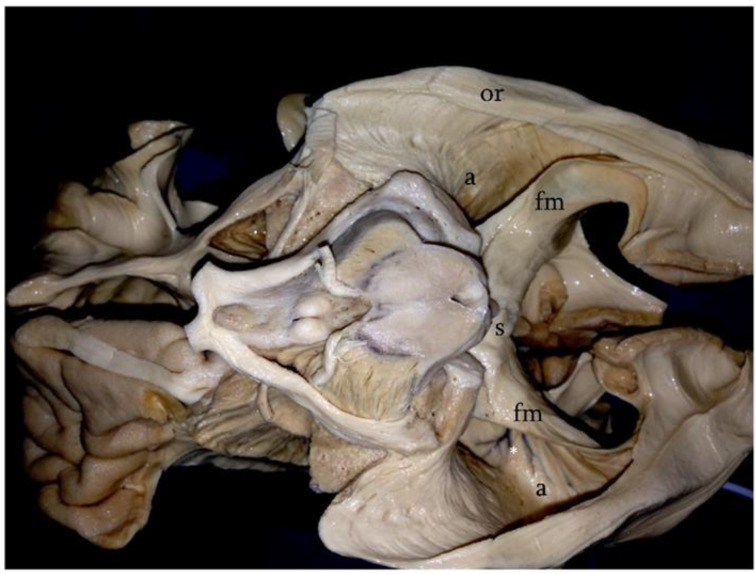
Klingler’s technique; basal surface of the cerebral hemispheres after the removal of the fusiform gyrus and inferior longitudinal fasciculus. In this photograph, the relationships between the atrial cavity (a) and the white matter fibers of the region can be observed. The lateral wall of the atrium is entirely covered by the optic radiations (or). Medially, the splenial fibers (s) form the forceps major (fm). On the left side, note the ependymal veins identifying the atrial cavity (asterisk).

Surgical removal of tumors of the atrium is a challenge, even to the most seasoned neurosurgeons. The issues are related not only to the deep location of this portion of the lateral ventricles but also to the vascularization of lesions of this area and to their complex three-dimensional relationships with the highly functional white matter fibers of the region [1,2,3]. In fact, the lateral wall of the atrium is entirely covered by a complicated system of fibers known as the sagittal stratum, which includes the optic radiations and some contingents of parietopontine and occipitopontine fibers. The basal surface is covered by the inferior longitudinal fasciculus, whereas white matter fibers related to the medial surface are the cingulum and the forceps major [4,5,6,7,8,9] (Figure 1). Given the functional importance of these structures and the often-benign nature of the lesions involving this area, an efficient approach to the atrium should be able to combine a total resection with respect to the anatomo-functional integrity of the white matter fibers [10]. Bearing this concept in mind, several surgical approaches to the atrium have been proposed [4]. The ipsilateral interhemispheric transprecuneus (IITA) approach satisfies these requisites. In this work, we present a clinical series of patients affected by tumors of the atrium that were approached through this elegant and complicated route. A detailed description of the technical aspects of the approach and a comparison with the possible alternatives is also given.

## 2. Methods

All patients who had undergone a surgical treatment for intraventricular tumors between January 2008 and January 2017 were retrospectively reviewed with institutional review board approval. Informed consent to archive and process the patients’ data was also obtained in an anonymous form. Patients’ demographics, clinical and radiological features, surgical approaches, and the immediate postoperative and final outcomes were retrieved using chart review. Histological findings and the involvement of the temporal horn are also reported. All patients underwent a pre-operative angio-MRI in order to study the localization of the bridging veins. In each patient, the choice of the surgical approach was critically evaluated according to anatomo-radiological features, including anatomy of the bridging veins, side of the lesion, vascularization, tumor characteristics, and the possible extension into the temporal horn. All patients were admitted to the intensive care unit after surgery for at least 24 h. Postoperative MRI studies were evaluated to assess the extent of resection and the existence of postoperative surgery-related complications. The follow-up was at least 3 years after surgery.

All procedures performed in the studies involving human participants were in accordance with the ethical standards of the institutional and/or national research committee and with the 1964 Helsinki Declaration and its later amendments or comparable ethical standards.

### 2.1. The Ipsilateral Interhemispheric Transprecuneus Approach: Surgical Steps (IITA)

**Figure 2:**
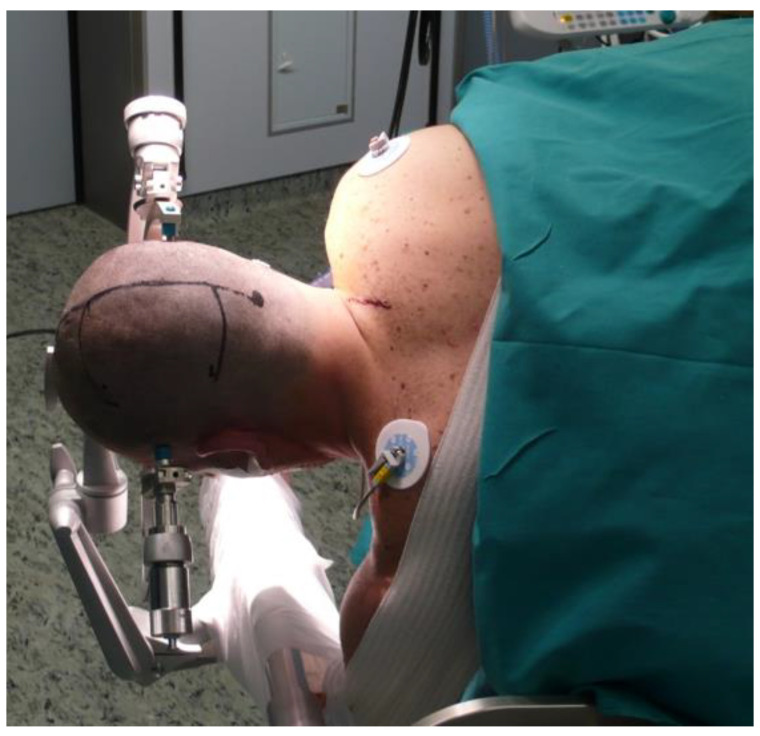
Patients were positioned in the three-quarter prone position with the head in the dependent oblique posture. The purpose of this maneuver is to obtain an enlargement of the interhemispheric window by inducing the cerebral hemisphere to fall away from the falx due to the effect of gravity. In this way, the need for cerebral retraction is significantly reduced, whereas the possibility to increase the angle of the “medial to lateral” dissection is augmented.

All patients underwent general anesthesia. All the procedures were performed by the senior author (PC). Before final positioning, a lumbar drain was placed to ensure adequate brain relaxation in all patients. Patients were placed in the standard three-quarter prone position with their head fixed in a 3-pin Mayfield head clamp in order to place the ipsilateral mastoid surface at the highest point of the surgical field. This can be obtained by combining the flexion of the head with a 15–20 degree rotation contralateral to the lesion and a latero-flexion towards the opposite side (Figure 2). The purpose of this maneuver is to obtain an enlargement of the interhemispheric window by inducing the cerebral hemisphere to fall away from the falx due to the effect of gravity. In this way, the need for cerebral retraction is significantly reduced, whereas the possibility to increase the angle of the “medial to lateral” dissection is augmented. Since June 2010, the Neuronavigation system (Stealth-Station AxiEM, Medtronic) and intraoperative monitoring of motor- and somatosensory-evoked potentials were systematically used. A linear or horseshoe parieto-occipital incision is performed. The incision should expose the sagittal as well as the lambdoid suture. Mannitol was administered at this time.

A parieto-occipital craniotomy centered on the superior sagittal sinus is performed. The dura mater is incised in a curvilinear fashion and reflected over the superior sagittal sinus. The interhemispheric fissure is opened, and the dissection is carried on through the gentle use of a fine aspirator, bipolar forceps, and cottonoids. Once the interhemispheric space has been gained, the first structure to recognize is the parieto-occipital artery running within the parieto-occipital sulcus. It represents the posterior limit of the precuneus. The anterior margin is constituted by the marginal ramus of the cingulate sulcus.

By following the parieto-occipital sulcus in the depth, the cingulate gyrus is encountered. It is limited by the cingulate sulcus above and the callosal sulcus below. It is recognizable by its whitish appearance and its perpendicular orientation with respect to the parieto-occipital sulcus.

At this point, 20 to 25 mL of cerebrospinal fluid (CSF) is released from the lumbar drain.

**Figure 3:**
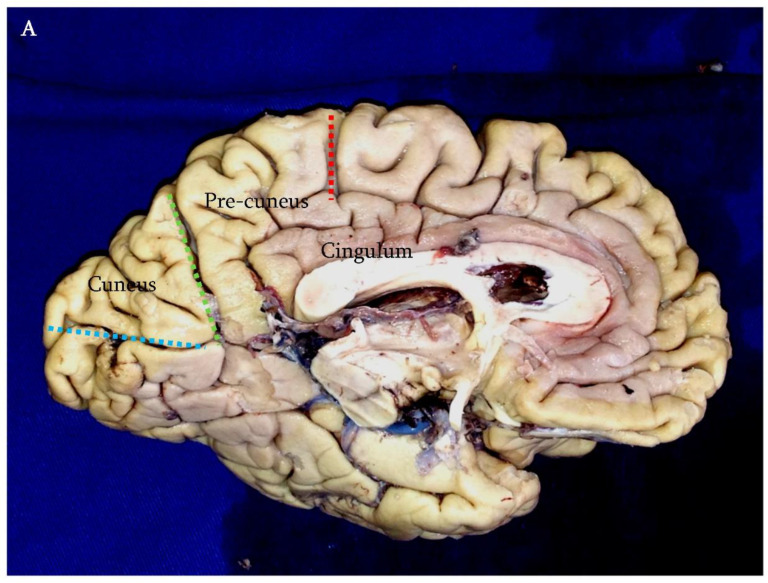

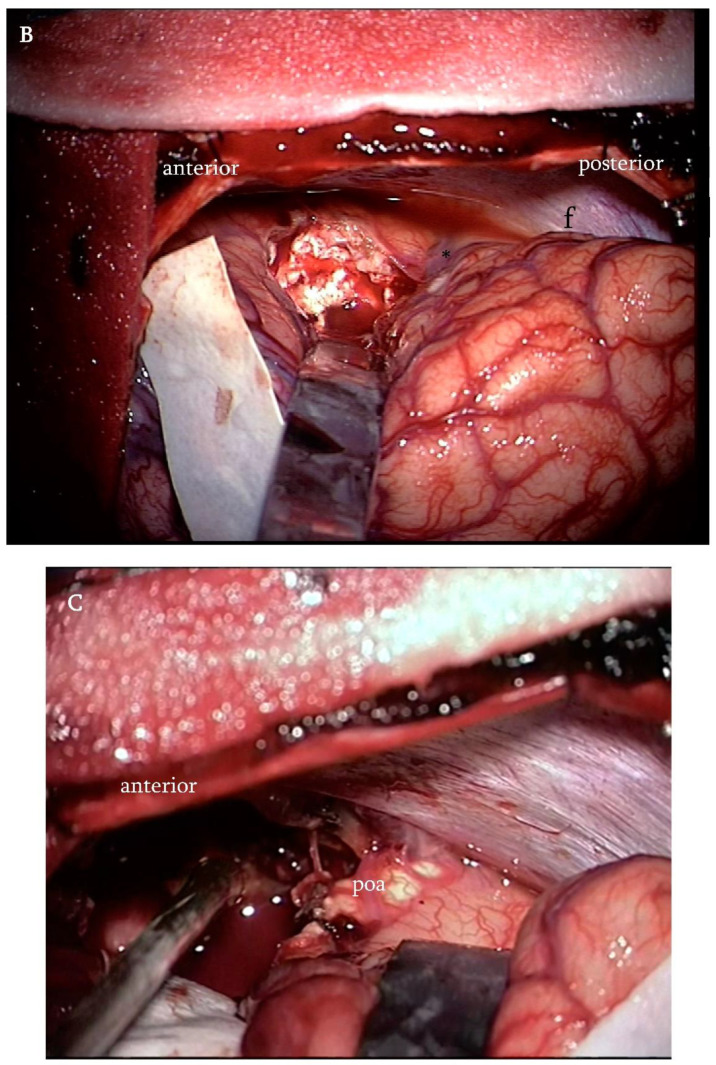
(**A**) Anatomic landmarks of the medial surface of the cerebral hemispheres. The precuneus is limited by the marginal ramus of the cingulate sulcus anteriorly (red dashed line) and by the parieto-occipital sulcus posteriorly (green dashed line). The cuneus comprises the area between the parieto-occipital sulcus and the calcarine sulcus (blue dashed line). In the IITA, the corticotomy (white circle) is performed just in front the deepest portion of the parieto-occipital sulcus, adjacent to the cingulum. (**B**) Intraoperative photographs showing the site of the corticotomy just in front of the parieto-occipital sulcus (asterisk) (f: falx). (**C**) The parieto-occipital artery (poa) identifying the parieto-occipital sulcus.

A 10 to 20 mm corticotomy is performed just in front of the inner portion of the parieto-occipital sulcus adjacent to the cingulate gyrus but without violating it under microscopic magnification. Brain retractors are placed. Gentle spreading of the margin of the corticotomy opens the corridor toward the atrial cavity. (Figure 3).

### 2.2. Data Collection Quality Assessment

Removal of the lesion was obtained by paying special attention with respect to the dissection plane between the lesion and the ependymal layer. On the medial wall, it is important to identify the choroid glomus and the calcar avis. The latter approximates the location of the calcarine fissure. Neuronavigation could help in the identification of these structures. Extra-axial lesions were internally debulked by Ultrasonic aspirator (CUSA Excel, Integra LifeSciences, Plainsboro, New Jersey), initially set at low aspiration and fragmentation power (25–30%) and high tissue selectivity (3–4) and subsequently modified according to the tumor’s characteristics.

## 3. Results

Between January 2008 and January 2017, 35 patients underwent surgical treatment for intraventricular tumors. Lesions were located in the atrium of the lateral ventricles in ten patients, and seven of these lesions were removed through the IITA.

Table 1 summarizes the patients’ characteristics, their clinical presentation, and histological and surgical findings. Ten patients (seven female and three male; female/male ratio 2.3/1) affected by lesions of the atrium of the lateral ventricle underwent surgical treatment, seven of which were approached through the ipsilateral interhemispheric approach (five women and two men; female/male 2.5/1). The mean age at the time of surgery was 42.8 years (range 6–63 years). Nine patients (90%) complained of severe, drug-resistant headaches, five patients (50%) showed lateral homonymous hemianopsia in the visual field, three patients (30%) had seizures, three patients (30%) harboring a lesion in the dominant hemisphere had speech disturbances, and one patient came in a comatose state because of the intralesional bleeding.

Seven patients harbored a meningioma (70%), 1 patient had a metastasis (10%), one patient had a choroid plexus papilloma (10%) and one patient had a cavernoma (10%). Five lesions (50%) were located in the dominant hemisphere, whereas the remaining five (50%) were non-dominant. In seven cases (70%), the lesion was purely intra-atrial, whereas in three cases (30%), an involvement of the temporal horn was documented. The tumor did not extend beyond the posterior third of the length of the temporal horn in any of these last cases.

In seven cases (70%), the ipsilateral interhemispheric transprecuneus approach was used. In two cases (20%), the lesion was removed through a transtemporal approach. In one case, a trans-superior parietal lobule approach route was chosen. In all the cases (100%), it was possible to obtain a gross total removal of the lesion.

At discharge, all the patients indicated a significant improvement of their headaches. At 3-year follow-ups, not one of the patients of this series complained of headache. Two patients experienced a worsening of the pre-operative visual disturbances. In one case, the worsening was permanent, whereas in the other case, the worsening was transient. Two patients experienced a significant improvement of their pre-operative visual symptoms. One patient remained stable. No patient who did not complain of visual disturbances pre-operatively described a post-operative worsening of visual symptoms.

All the patients who presented with seizures were seizure-free at the 3-year follow-up. Speech disturbances completely disappeared in all the symptomatic patients for this disturbance. One patient experienced hydrocephalus and required a ventriculo-periotoneal shunt. One patient experienced a transient memory impairment. One patient had transient visuo-spatial disturbances. At the 3-year follow-ups, no patient had experienced a pathology recurrence.

### Case Presentation

**Figure 4:**
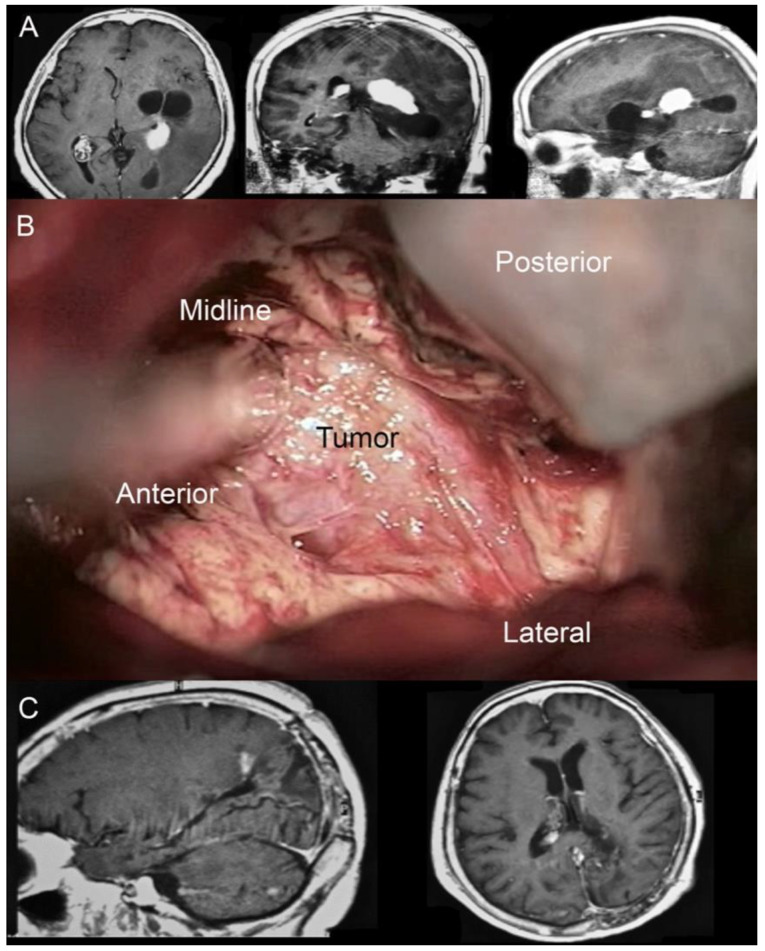
(**A**) Pre-operative MRI showing a left-sided partially cystic lesion localized in the atrium and partially involving the temporal horn. (**B**) Intraoperative photograph propaedeutic for videoclip orientation. (**C**) Postoperative MRI showing complete tumor removal.

- Illustrative case n.1 (Case n.2 in the table, Figure 4)

A 65-year-old female with a clinical history characterized by a slow and progressive reduction of the visual field and chronic headaches came to our attention because of an episode of seizure. A brain MRI showed a left-sided partially cystic lesion localized in the atrium and partially involving the temporal horn. Upon neurological examination, she was observed to have a lateral homonymous hemianopsia. She had normal motor strength in all extremities and no speech disturbances. She was right-handed. The patient underwent surgical removal of the lesion through IITA. Once the interhemispheric window was gained and the parieto-occipital sulcus identified, a 1 cm corticotomy perpendicular to the deepest end of the sulcus and adjacent to the cingulum was performed. Once the atrial cavity was entered, the lesion was identified. Its consistency was very hard. A patient and delicate dissection of the lesion from the surrounding ventricular ependyma was performed by the wise use of suction devices, bipolar forceps, and micro-dissectors. Once the tumor/brain interface was identified all around the lesion’s circumference, the lesion itself was separated into two pieces through the use of the Malis bipolar forceps. The biggest part of the tumor was removed by gently completing the dissection from the surrounding ependyma. Removal of the remnant portion was obtained by repeating the maneuvers used for the biggest portion.

The postoperative course was uneventful. The patient was admitted to the neurocritical care unit, where she was monitored for 24 h. The patient experienced a significant improvement of the visual field disturbances documented by campimetry, and her headache slowly recovered. At a 3-year follow-up, no seizures had been documented. Histological examination showed a choroid plexus papilloma.

**Figure 5:**
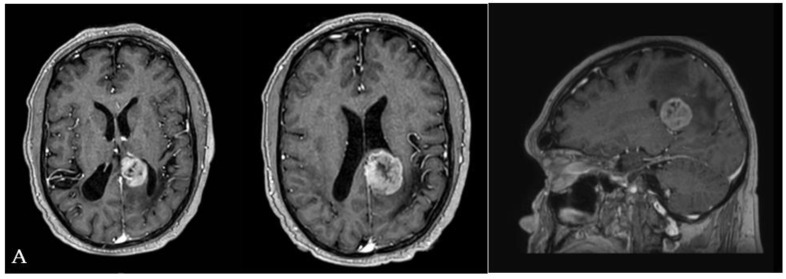

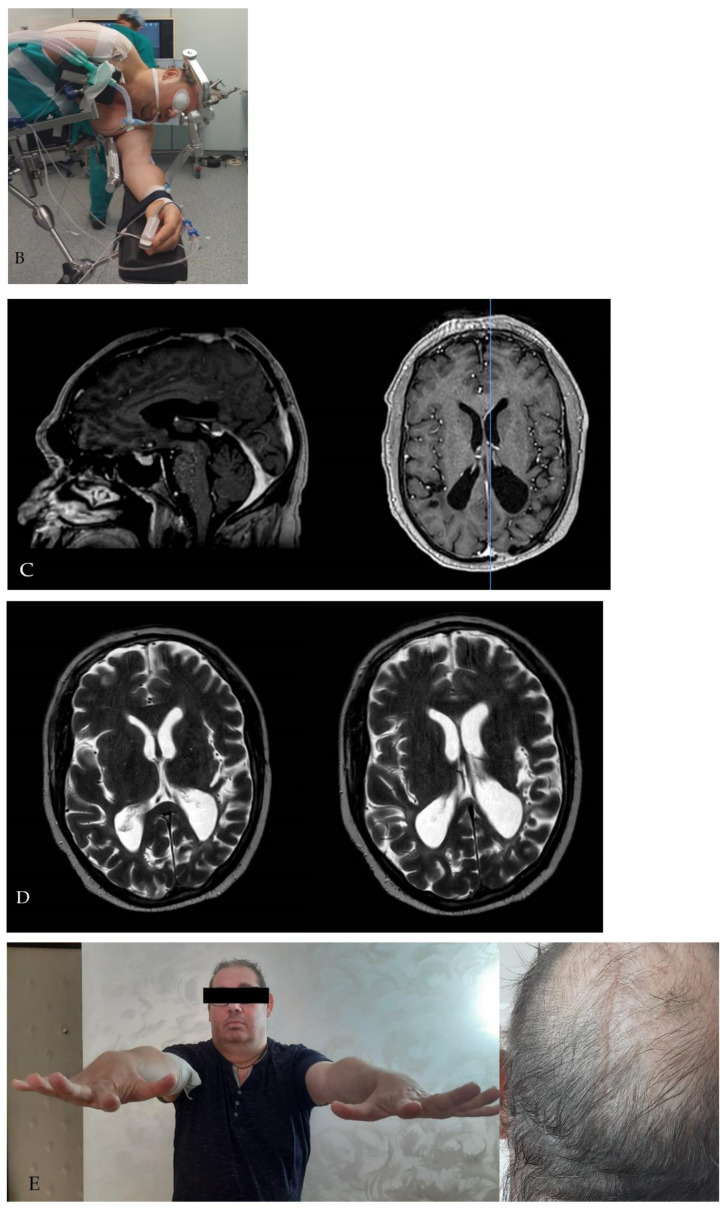
(**A**) Pre-operative MRI documenting the presence of a lesion partially involving the left atrial cavity of the lateral ventricle. (**B**) Three-quarter prone position for the IITA. (**C**) Immediate post-operative MRI (T1 sequence with cm) control documenting the complete resection of the lesion and the rehabilitation of the atrial cavity. (**D**) Immediate post-operative MRI (T2 sequence). Note the absence of post-surgical brain edema. (**E**) One month post-operative neuological evaluation and surgical linear incision aspect.

- Illustrative case n.2 (Case n.4 in the table, Figure 5)

A 35-year-old man with a history of melanoma came to our attention because of a drug-resistant headache and aphasia. A brain MRI was performed documenting the presence of a left-sided solid lesion enhancing after the administration of mc partially involving the atrial cavity of the lateral ventricle. The total body CT scan excluded the presence of other metastases. The patient underwent surgical removal through IITA.

Immediately, a post-operative MRI was performed, documenting the complete removal of the lesion. T2 weighted sequences excluded the presence of post-surgical brain edema.

The post-operative course was favorable. His aphasia was remitted, as well as his headache. The patient was discharged on the fifth post-operative day.

## 4. Discussion

Surgery of tumors located within the atrium is challenging. The main problems that neurosurgeons are called to face are represented by the highly functional relevance of the cortex, white matter fibers, and arteries located in this area of the brain. [5,11].

Moreover, the atrium is entirely encircled by a highly functional system of white matter fibers that should be preserved during the surgical removal of tumors of this area. The main systems of fibers encountered while approaching the atrium are the optic radiation covering its lateral surface, the inferior longitudinal fasciculus extending along its base, and the splenial fibers together with the cingulum on the medial side [12]. All these systems of fibers play important and vital neurological functions. Moreover, in dominant hemispheres, the lateral wall of the atrium is in a close relationship with the system of fibers connecting the language areas [13,14].

Given these premises, it is understandable how generations of neurosurgeons have focused their attention on the study of surgical approaches to the atrium that are capable of conjugating the tumor resection with the preservation of the anatomo-functional integrity of these intricate and complex systems of white matter fibers [15,16].

With this purpose, several surgical approaches have been proposed [6,17,18]. Each of these has its advantages and disadvantages, as well as specific indications depending on the tumor characteristics, localization of the lesion within the ventricular system (purely atrial vs. tumors extending within or outside the ventricular system), hemispheric dominance, the patient’s symptoms, and the neurosurgeon’s experience [19,20].

On the base of the surgical route chosen, approaches can be subdivided into different categories:

- Trans-sylvian approach;
- Trans-cortical approaches (trans-temporal and trans-superior parietal lobule approach);
- Subtemporal approach;
- Posterior interhemispheric approaches (trans-splenium, ipsilateral interhemispheric trans-precuneus approach; contralateral transfalcine transprecuneus approach);
- Infra-transtentorial approaches.

The distal trans-sylvian approach is a challenging approach indicated for the treatment of small lesions involving the atrium. It requires an extensive and technically demanding dissection of the Sylvian fissure and identification of the Heschl gyrus. By following the long axis of the latter, the atrium can be reached. The approach cannot be used in dominant hemispheres, given the risk of damaging the language areas [21]. Transcortical approaches include the trans-temporal route and the superior parietal lobule approach.

These approaches were used in the first years of our experience and were abandoned because of the high risk of complications.

The trans-temporal approach has its theoretical indications for the treatment of lesions involving the antero-inferior portion of the atrium and the posterior third of the temporal horn. Its main limit is the unavoidable damage to the optic radiations. Moreover, it cannot be used in dominant hemispheres [22]. In our series, two patients were operated on with this route. Both were affected with lesions located in non-dominant hemisphere and presented campimetric disturbances before surgery. In both cases, the patients experienced a worsening of neurologic conditions with a reduction of the campimetry. In one case, the worsening was transient and improved with steroids, whereas in the other one, the deficit was permanent.

The superior parietal lobule approach was used only once in our series for the treatment of a non-dominant meningioma in which the atrium was displaced cranially. Indications for this approach are lesions localized in the atrium and partially involving the cella media. The main risks/disadvantages of this approach are represented by the risk of visual field disturbances, speech and agnostic disturbances in dominant hemispheres, and visuo-spatial alteration in non-dominant hemispheres.

Our patient harbored a right-sided, purely intra-atrial meningioma and experienced transient a visuospatial syndrome characterized by writing and written math calculations.

In our series, we never used the subtemporal approach since we considered this route unfavorable in terms of tumor exposition, surgical comfort, and risk of complication (temporal lobe retraction, brain swelling, and venous damage).

Similarly, we did not have the possibility to test the efficacy of the infratentorial supracerebellar approach nor the transtentorial approach in the removal of intra-atrial lesions. We consider the indication for these approaches too limited (impossibility to expose the superior portion of the atrium and difficulty controlling lesions not strictly localized in the midline) and its risks/disadvantages (long working distance, risk of damage to the bridging veins and venous sinuses, and cerebellar swelling) too elevated [23].

The ipsilateral interhemispheric transprecuneus approach was widely used in our series with encouraging results. Despite the limitations related to the relatively rarity as a clinical entity as well as the low number of patients of this series, the results of our experience with the IITA are encouraging. In particular, in all the patients treated with this approach, we experienced a complete resolution of pre-operative symptoms or at least a significant improvement of their conditions. In particular, patients affected with visual field impairments indicated a recovery of their deficit (complete in one case, partial in the other one, both cases confirmed by campimetry). Moreover, seizures and speech disturbances reversed after surgical treatment. The only complications encountered in our series were one case of transient memory impairment and one case of hydrocephalus.

The first case was probably related to the fact that the lesion was a second-grade meningioma according to the WHO classification in which the dissection plane between the tumor and the surrounding brain parenchyma was not clearly identifiable. In the post-operative course, the CT scan documented a brain edema partially involving the cingulum. The disturbance spontaneously recovered after 2 weeks.

The hydrocephalus occurred in a six-year-old female who came to our attention in a comatose state because of a bleeding cavernoma causing an intraventricular hemorrhage. The cause of hydrocephalus must be attributed to the hemorrhage and the treatment through a ventriculo-peritoneal shunt was resolutive.

The ipsilateral interhemispheric transprecuneus approach (IITA) was first described by Yasargil [24]. The main advantage of this route is that by exploiting a natural pathway as the interhemispheric fissure, it permits the neurosurgeon to reach the atrium while preserving the anatomo-functional integrity of the white matter fibers covering the atrial walls [24].

The main disadvantages attributed to this route are the deep and narrow working window, the difficulty of exposing the lateral portion of the lesion, the presence of bridging veins directed to the superior sagittal sinus, brain retraction, and the risk of memory impairment in case of incision of the cingulum [24].

A theoretical resolution to the reduced angle of surgical exposure attributed to IITA has been found by the proposal of the interhemispheric transfalx transprecuneus approach as a possible alternative. Authors attribute to this route the advantages of an increased working angle, a better and easier visualization of the lateral portion of the lesion, and a reduced need for brain retraction. This latter aspect could contribute to an increase in the accuracy of the neuronavigation system [25].

Our experience with IITA prompts us to state that in this approach, the key to increasing the angle exposure of the lateral wall of the atrium is the correct head positioning. In fact, the three-quarter prone position with the head in the dependent oblique posture toward the side of the lesion enlarges the interhemispheric space by inducing the cerebellum to fall way from the falx due to the effect of gravity. This aspect, coupled with the deliquoration induced by the interhemispheric dissection, limits the need for brain retraction and increases the angle of surgical exposure facilitating the visualization of the lateral wall of the atrium. Moreover, this position places the axis of the lesion in a nearly perpendicular plane with respect to the working angle, increasing the operative comfort for the surgeon.

The keys for success in this approach are represented by a wide opening of the interhemispheric fissure, adequate deliquoration, and correct positioning of the patient. All these aspects significantly limit the necessity for brain retraction and the consequent possible damages related to it (brain infarction, brain edema, etc.)

In our experience, we did not find neuronavigation essential in this approach. In fact, this is an anatomic route in which the guide for a proper intraoperative orientation should be attributed to the identification of anatomic landmarks rather than to the neuronavigation system. In particular, in our series, we were able to easily identify the parieto-occipital artery in all the procedures performed. The recognition of this anatomic landmark is pivotal since it runs within the parieto-occipital sulcus and consequently facilitates the identification of the precuneus limits.

Regarding the risk of memory impairment, we had only one transient disturbance. Moreover, as mentioned above, the memory impairment was not attributable to the incision of the cingulum but to post-operative edema after the removal of a not-easily detachable grade II meningioma. In our series, we always identified the cingulum before performing the corticotomy in order to avoid its damage. It can be easily recognized because of its whitish color and its orientation perpendicular to the parieto-occipital sulcus. The corticotomy was always performed at the deepest end of the pre-cuneus, perpendicular to the parieto-occipital sulcus and adjacent to the cingulum. The length of the corticotomy exceeded 1 cm only in those cases in which the lesion involved the posterior third of the temporal horn.

Concerning the limits related to the presence of bridging veins, the simple execution of a pre-operative angio-MRI is sufficient to select those cases in which the interhemispheric approach could not be performed. In our series, not one of the approaches had to be changed because of the patient’s venous anatomy.

## 5. Conclusions

Surgical treatment of tumors of the atrium is challenging because of the need to conjugate a total removal of the lesion with respect to the anatomo-functional integrity of the white matter fibers encircling its walls. The IITA was successfully used in seven patients, permitting us to obtain a total removal of the lesion in all the cases treated without experiencing postoperative neurologic deficits related to white matter fiber injuries. The key to the successful execution of this approach is the correct positioning of the patient. The dependent oblique posture in the three-quarter prone position facilitates the enlargement of the interhemispheric fissure, increasing the working angle and facilitating the exposure of the lateral wall of the atrium.

## Figures and Tables

**Table 1:** Demographic information, presenting symptoms, histology, and outcomes of patients affected by lesions of the atrium of the lateral ventricle. TH: temporal horn; IITA: ipsilateral interhemispheric transprecuneus; GRT: gross total removal; VPS: ventriculoperitoneal shunt.

Patient	Age	Sex	Clinical Presentation	Tumor Location	Surgical Approach	Histology	Extent of Resection	Complications
1	6	F	Coma	Atrium	IITA	Cavernoma	GRT	Hydrocephalus requiring VPS
2	65	F	Headache + seizures + lateral homonymous hemianopsia	Partial involvement TH	IITA	Chorid plexus papilloma	GRT	None
3	48	F	Headache	Atrium	IITA	Meningioma	GRT	None
4	35	M	Headache + aphasia	Atrium	IITA	Metastases	GRT	None
5	60	F	Headache + aphasia	Atrium	IITA	Meningioma	GRT	Transient memory impairment
6	21	F	Headache + lateral homonymous hemianopsia	Partial involvement TH	IITA	Meningioma	GRT	None
7	63	F	Headache + seizures + aphasia	Atrium	IITA	Meningioma	GRT	None
8	44	M	Headache + lateral homonymous hemianopsia	Atrium	Transtemporal	Meningioma	GRT	None
9	47	M	Headache + seizures+ lateral homonymous hemianopsia	Partial involvement TH	Transtemporal	Meningioma	GRT	None
10	39	F	Headache + lateral homonymous hemianopsia	Atrium	Trans-superior parietal lobule	Meningioma	GRT	Transient visuo-spatial alteration

## Data Availability

Data sharing not applicable.
